# Population structure and antibiotic resistance profiles of *Mycobacterium tuberculosis* isolates from Ibadan, Nigeria (2019–2020): a pilot study to improve affordable molecular diagnostic tools

**DOI:** 10.3389/fpubh.2025.1657825

**Published:** 2025-09-29

**Authors:** Elsy Carvajal, Samantha Escandón, Pelumi Daniel Adewole, Bernardo Castro-Rodríguez, Ángel Sebastián Rodríguez-Pazmiño, Solon Alberto Orlando, Alexandra Narvaez, Olumuyiwa Samuel Alabi, Miguel Angel Garcia-Bereguiain

**Affiliations:** ^1^One Health Research Group, Universidad de Las Américas, Quito, Ecuador; ^2^Department of Pharmaceutical Microbiology, Faculty of Pharmacy, University of Ibadan, Ibadan, Nigeria; ^3^Instituto Nacional de Salud Pública e Investigación, Guayaquil, Ecuador; ^4^Universidad Ecotec, Guayaquil, Ecuador; ^5^Universidad Espíritu Santo, Guayaquil, Ecuador

**Keywords:** Nigeria, *Mycobacterium tuberculosis*, antibiotic resistance, MIRU-VNTR, sublineages

## Abstract

Nigeria ranks as the sixth country globally and the first in Africa with the highest burden of tuberculosis (TB) infection. The emergence and spread of multidrug-resistant TB (MDR-TB) strains have posed significant challenges to effective disease management in the country. In this study, 55 *Mycobacterium tuberculosis* (MTB) isolates from patients attending a hospital in Ibadan city (Nigeria) were selected. MTB isolates were analyzed using PCR amplification of gene fragments associated with antibiotic resistance, followed by Sanger sequencing and bioinformatics analysis. Additionally, MIRU-VNTR genotyping was performed to address population structure and transmission dynamics. Results show an association between mutations in the *rpoB*, *inhA* and *gyrA* genes and phenotypic resistance to rifampicin, isoniazide and fluoroquinolones in a significant percentage of the MTB isolates. However, an extended panel of genes would enable a better characterization of antibiotic resistance. The population structure of MTB in Ibadan, as determined by using MIRU-VNTR, revealed that 96.1% of the strains belong to lineage 4, distributed in the following sublineages: Uganda I (47.1%), LAM (21.6%), Cameroon (17.6%), and Ghana (9.8%). Meanwhile, 3.9% of the strains correspond to lineage 5 (L5), West African-1 sub-lineage. The population structure was very heterogeneous and no active transmission clusters were detected. Overall, this pilot study demonstrated the utility of cost-effective molecular tools in enhancing TB surveillance and control programs in settings where whole-genome sequencing (WGS) is still an economical challenge.

## Introduction

Nigeria is one of the most densely populated countries in West Africa, where significant social and economic inequalities contribute to inadequate healthcare coverage and inequitable access to medical services (1). Data indicate that less than 3% of Nigerians are enrolled in the National Health Insurance Scheme (NHIS), leaving 97% of the population, including vulnerable and disadvantaged groups, without access to this benefit (2). According to the World Health Organization (WHO), Nigeria ranks as a high-burden country for TB, placing sixth globally and first in Africa. In 2023, the WHO reported a total of 499,000 TB incidence cases, representing a 16% increase compared to the 418,000 cases recorded in 2015 (3). The increase in cases has exacerbated the TB crisis in the country, due to the emergence and spread of drug-resistant (DR-TB) and multidrug-resistant (MDR-TB) strains, posing a major public health challenge (4).

MDR-TB is characterized by resistance to isoniazid and rifampicin, the two most potent first-line anti-TB drugs. In 2023, Nigeria reported an estimated 9,400 cases of MDR-TB or rifampicin-resistant tuberculosis (RR-TB) (3). Alarmingly, 57% of individuals with bacteriologically confirmed TB that year exhibited resistance to both rifampicin and fluoroquinolones, meeting the criteria for pre-XDR-TB or XDR-TB (5). These figures highlight the critical severity of MDR-TB in Nigeria, emphasizing the urgent need for more effective strategies in diagnosis, treatment, and prevention. The situation is further exacerbated by poor adherence to anti-TB programs, widespread self-medication, and challenges in completing treatment due to the high cost and frequent shortages of anti-TB drugs (6).

The alarming rise of MDR-TB and XDR-TB in Nigeria is driven by the accumulation of spontaneous mutations in *Mycobacterium tuberculosis* (MTB), which lead to significant phenotypic changes, particularly in antibiotic resistance. Mutations in key genes such as *rpoB*, *katG,* and *gyrA* play a crucial role in the bacterium’s ability to evade antimicrobial treatments. For instance, mutations in *rpoB* alter RNA polymerase, reducing its affinity for rifampicin and leading to resistance. Similarly, mutations in *katG* prevent the activation of isoniazid, one of the most effective anti-TB drugs. Meanwhile, mutations in the promoter region of *inhA* reduce the drug’s ability to inhibit the enoyl-ACP reductase enzyme, further contributing to resistance to isoniazid. Additionally, mutations in *gyrA*, which encode a subunit of DNA gyrase, confer resistance to fluoroquinolones, an important second-line treatment. Beyond conferring drug resistance, these genetic adaptations enhance the bacterium’s survival and persistence in diverse environments, further complicating TB control and eradication efforts (7, 8). According to the literature, the following regions in the *rpoB*, *katG,* and *gyrA* genes are considered hotspots for mutations associated with antibiotics resistance, particularly within the rifampin-resistance determining region (RR-DR) between codons 507 and 533 (9–11). In *katG*, mutations (particularly at codon 315) can lead to isoniazid resistance by impairing the activation of the drug (10, 11). The *gyrA* gene, which encodes the A subunit of DNA gyrase, frequently exhibits mutations in its quinolone-resistance determining region, resulting in fluoroquinolone resistance (9). These genetic changes highlight the complexity of resistance mechanisms and emphasize the importance of monitoring these loci in clinical and diagnostic settings (11). These genetic changes not only confer resistance but also influence the bacterium’s adaptability and survival under selective pressures, complicating eradication efforts. Monitoring these genetic loci is crucial for the early detection and effective management of drug-resistant tuberculosis (11). Given the increasing prevalence of MDR and XDR-TB strains, understanding the molecular basis of resistance is imperative for the development of innovative diagnostic tools and treatment strategies (11).

Molecular epidemiology utilizes molecular techniques to analyze the population structure and transmission dynamics of MTB in a particular setting. Currently, various molecular and sequencing methodologies are available for epidemiological studies, including single-nucleotide polymorphism (SNPs) analysis, mycobacterial interspersed repetitive unit variable number tandem repeats (MIRU-VNTR), and whole genome sequencing (WGS) (12). Most of these tools are reproducible and provide a more comprehensive view of epidemiological studies, highlighting WGS as the molecular characterization technique with the most significant potential for discrimination in the diagnosis of *M. tuberculosis* (13). MIRU-VNTR is structured with 40-100-bp repetitive sequences organized in direct tandem repeats across various loci within the chromosome of MTB H37Rv (with 41 loci present). Functioning akin to minisatellites, these sequences exhibit 12 loci MIRUs that demonstrate variations in tandem repeat copy numbers and sequence variations between repeats (14). The method involves PCR amplification followed by gel electrophoresis, making it a robust and straightforward tool for genotyping. MIRU-VNTR stands out due to its simplicity high reproducibility, cost-effectiveness, and ability to identify species within the *Mycobacterium tuberculosis* complex (MTBC) and their genotypes, making it particularly accessible for developing countries and facilitating easy integration into a global database for strain comparison worldwide^1^ (15).

This study aimed to analyze the population structure and mutation profiles of the *rpoB*, *inhA*, *katG*, and *gyrA* genes in MTB isolates from patients attending a hospital facility in Ibadan, Nigeria, during 2019 and 2020.

## Materials and methods

### Study population and setting

The study was conducted at the Government Chest Hospital in Ibadan, Oyo State, Nigeria, between December 2019 and October 2020, and involved MTB culture from pulmonary tuberculosis patients. The inclusion criteria were that participants had to consent to be included in the study. No personal information about the patients was collected for this study, and further analysis was focused on MTB cultures isolated from these patients.

### MTB culture and drug susceptibility testing

MTB culture and drug susceptibility (first- and second-line drugs) testing from sputum samples was carried out as described elsewhere (11, 16). A total number of 72 MTB isolates were collected during the study period and were sent from Ibadan to Universidad de Las Américas (Ecuador) for further analysis (Table 1).

**Table 1:** Drug susceptibility testing for MTB isolates included in the study.

No.	Sample	Susceptibility to anti-TB drugs: sensitivity (S) or resistance (R)										Phenotype
		RIF	INH	FQ	K	A	C	L	P	E	ET	
1	NGR2	R	S	S	S	S	S	S	S	S	S	RR-DR
2	NGR3	S	S	S	S	S	S	S	S	S	S	Sensible
3	NGR4	R	R	R	S	S	S	S	S	S	S	Pre-XDR
4	NGR7	R	S	S	S	S	S	S	S	S	S	RR-DR
5	NGR8	S	S	S	S	S	S	S	S	S	S	Sensible
6	NGR9	R	S	R	R	R	R	R	S	S	S	Pre-XDR
7	NGR10	R	S	S	S	S	S	S	S	S	S	RR-DR
8	NGR11	R	-	S	S	S	S	S	S	S	S	RR-DR
9	NGR13	R	S	S	S	S	S	S	S	S	S	RR-DR
10	NGR14	R	S	S	S	S	S	S	S	S	S	RR-DR
11	NGR15	R	S	S	S	S	S	S	S	S	S	RR-DR
12	NGR16	R	S	S	S	S	S	S	S	S	S	RR-DR
13	NGR17	R	S	S	S	S	S	S	S	S	S	RR-DR
14	NGR18	R	S	S	S	S	S	S	S	S	S	RR-DR
15	NGR19	R	S	R	R	R	R	R	R	S	S	Pre-XDR
16	NGR20	S	S	S	S	S	S	S	S	S	S	Sensible
17	NGR21	R	S	S	S	S	S	S	S	S	S	RR-DR
18	NGR22	S	S	S	S	S	S	S	S	S	S	Sensible
19	NGR23	S	S	S	S	S	S	S	S	S	S	Sensible
20	NGR24	S	S	S	S	S	S	S	S	S	S	Sensible
21	NGR25	S	S	S	S	S	S	S	S	S	S	Sensible
22	NGR26	S	S	S	S	S	S	S	S	S	S	Sensible
23	NGR27	S	S	S	S	S	S	S	S	S	S	Sensible
24	NGR28	R	S	S	S	S	S	S	S	S	S	RR-DR
25	NGR29	R	S	R	S	S	S	R	S	S	S	Pre-XDR
26	NGR30	S	S	S	S	S	S	S	S	S	S	Sensible
27	NGR31	S	S	S	S	S	S	S	S	S	S	Sensible
28	NGR32	R	R	R	R	R	R	R	S	S	S	Pre-XDR
29	NGR33	S	S	S	S	S	S	S	S	S	S	Sensible
30	NGR43	R	S	S	S	S	S	S	S	S	S	RR-DR
31	NGR44	R	S	S	S	S	S	S	S	S	S	RR-DR
32	NGR45	R	S	S	S	S	S	S	S	S	S	RR-DR
33	NGR46	S	S	S	S	S	S	S	S	S	S	Sensible
34	NGR47	R	S	S	S	S	S	S	S	S	S	RR-DR
35	NGR48	R	S	S	S	S	S	S	S	S	S	RR-DR
36	NGR50	R	R	S	S	S	S	S	S	S	S	MDR
37	NGR53	R	S	S	S	S	S	S	S	S	S	RR-DR
38	NGR55	R	S	S	S	S	S	S	S	S	S	RR-DR
39	NGR56	S	S	S	S	S	S	S	S	S	S	Sensible
40	NGR59	R	S	S	S	S	S	S	S	S	S	RR-DR
41	NGR60	S	S	S	S	S	S	S	S	S	S	Sensible
42	NGR61	R	R	S	S	S	S	S	S	S	S	MDR
43	NGR62	R	R	S	S	S	S	S	S	S	S	MDR
44	NGR63	R	R	S	S	S	S	S	S	S	S	MDR
45	NGR64	R	S	S	S	S	S	S	S	S	S	RR-DR
46	NGR65	R	S	S	S	S	S	S	S	S	S	RR-DR
47	NGR66	R	S	S	S	S	S	S	S	S	S	RR-DR
48	NGR67	R	R	S	S	S	S	S	S	S	S	MDR
49	NGR69	S	S	S	S	S	S	S	S	S	S	Sensible
50	NGR70	R	S	S	S	S	S	S	S	S	S	RR-DR
51	NGR71	R	S	S	S	S	S	S	S	S	S	RR-DR

### DNA extraction

Genomic DNA from MTB isolates was extracted using the boiling method (17). Briefly, MTB cultures were resuspended in TE buffer (10 mM Tris–HCl, 1 mM EDTA, pH 8.0), boiled at 95 °C for 45 min, and centrifuged at 10,000 g for 5 min. Supernatant was used directly for molecular assays. Of the 72 samples received from Ibadan, good quality DNA was recovered for 51 samples. Of those 51 samples, 35 MTB strains that were phenotypically resistant were included in further analysis for PCR and Sanger sequencing.

### PCR and sanger sequencing for *rpoB, inhA, katG, and gyrA* genes

Gene segments associated with antibiotic resistance were amplified using the primers listed in Supplementary Table 1.

For the *rpoB* gene (RR-DR), the PCR mixture contained DNA, 7.5 μL of 2X Gotaq Green Master Mix (Promega), 0.15 μL of each primer (0.13 μM), and 6.1 μL distilled water for a final volume of 15 μL. The cycling program consisted of 2 min at 94 °C, followed by 30 cycles of 45 s at 94 °C, 1 min at 65 °C, and 60 s at 72 °C, with a final elongation at 72 °C for 5 min during one cycle.

For the *inhA* and *katG* genes, the PCR mixture consisted of 7.5 μL of 2x Gotaq Green Master Mix (Promega), 1.5 μL of each primer (1 μM), 3.5 μL distilled water, in a final volume of 15 μL. The cycling program consisted of 2 min at 94 °C, followed by 30 cycles of 45 s at 94 °C, 1 min at 65 °C, 1 min at 72 °C, with a final elongation at 72 °C for 5 min during one cycle.

For the *gyrA* gene, the PCR mixture contained 7.5 μL of 2X Gotaq Green Master Mix (Promega), 0.15 μL of each primer (0.4 μM), 5.3 μL distilled water, in a final volume of 15 μL. The cycling program was set at 5 min at 94 °C, followed by 35 cycles of 45 s at 94 °C, 30 s at 55 °C, and 50 s at 72 °C, with a final elongation at 72 °C for 10 min during one cycle.

PCR products were amplified in 2% agarose gels with a 100 bp ladder (ABM) to confirm amplification visually.

Sanger sequencing was performed at *Laboratorios de Investigación* of the Universidad de Las Américas (Quito, Ecuador), using the DNA Sequencing by Capillary Electrophoresis protocol described by ThermoFisher (18). The genetic analysis and the sequence alignment were performed using the software Geneious (version 2023.0.2), with the MTB H37Rv genome as the reference (accession number: NC_000962.3), downloaded from the NCBI Genome database.^2^

### MIRU-VNTR genotyping of MTB isolates

The MIRU-VNTR genotyping was conducted through PCR amplification of 12 loci followed by visualization in 2% agarose gels, as described previously. Genotyping data of the isolates were analyzed using the MIRU-VNTRplus web application available on: www.miru-vntrplus.org. Sublineage identification was performed by similarity search using MIRU-VNTR information through categorical distance measure (MIRU-VNTR weight: 1). The neighbor-joining tree (NJT) and calculation of Minimum Spanning Tree (MST) were also performed using MIRU-VNTR information.

## Results

### Mutation analysis of *rpoB, inhA, katG, and gyrA* genes

**Figure 1:**
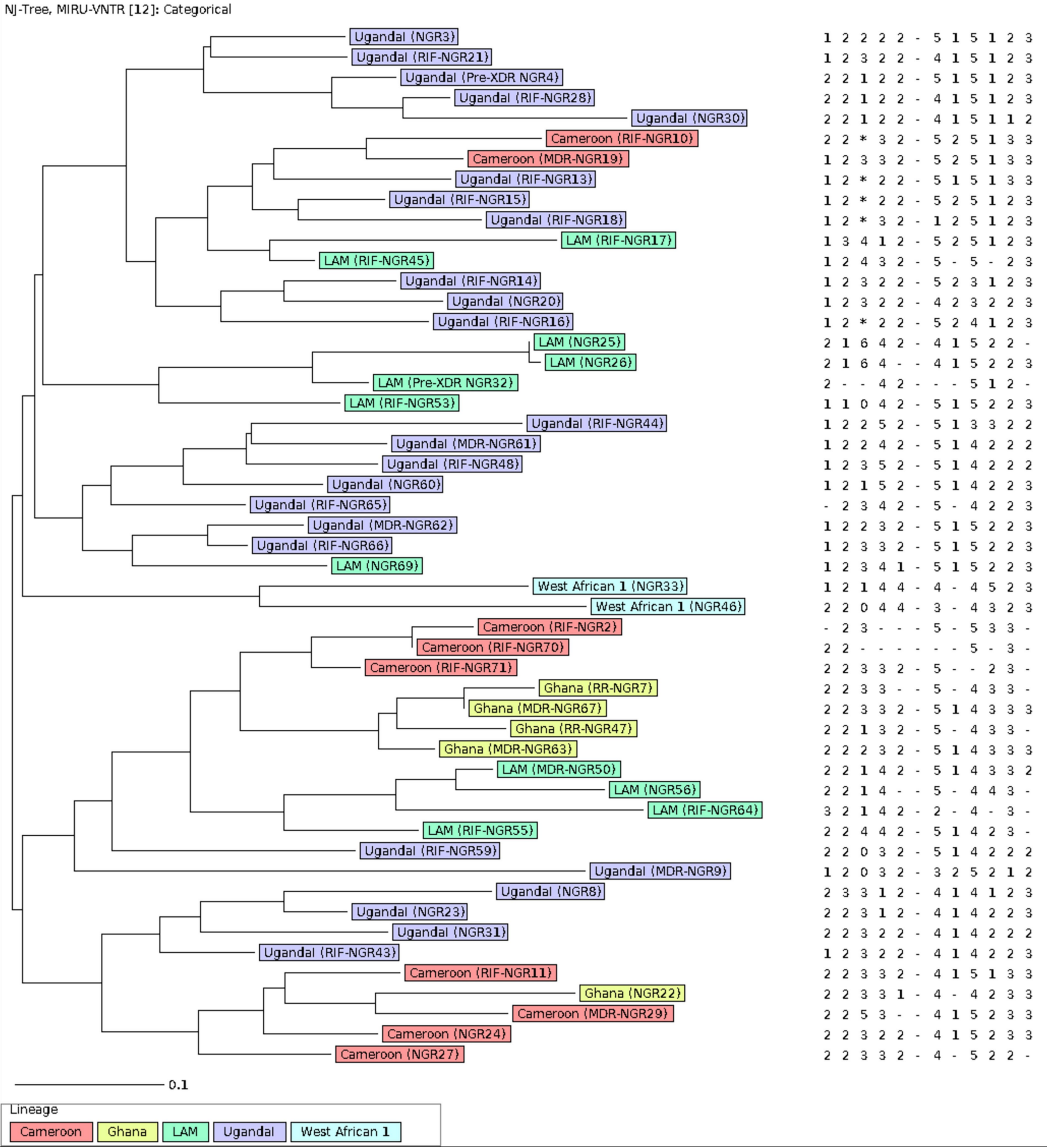
The population structure of 51 MTB strains from Ibadan Nigeria included in the study. The Neighbor-joining tree was constructed on 12 –loci MIRU-VNTR with default parameters. Each color represents a lineage. The label on the samples contains the resistance of each strain. The analysis was performed using the MIRU-VNTRplus web application.

The drug susceptibility testing results for the 51 MTB isolates with good DNA quality are detailed in Figure 1. There were 35 MTB isolates resistant to rifampicin, seven isolates resistant to isoniazid and five isolates resistant to fluoroquinolones.

Sanger sequencing results of the *rpoB* gene for the 35 MTB strains resistant to rifampicin are showed in Table 2. Twelve MTB strains (34.3%) were discarded due to poor sequencing quality. For the remaining 23 MTB strains, 10 (43.5%) had mutations in the RR-DR region of the *rpoB* gene. Mutations in codon 531 were found in 7 strains, while mutations in codons 513, 526 and 533 were found in one single MTB isolate each (Table 2). In 13 MTB strains (56.5%) that were phenotypically rifampicin-resistant, there were no mutations in the RD-DR region.

**Table 2:** Mutations analysis of *rpoB, katG, inhA*, and *gyrA* for the MTB isolates included in the study.

Gene	Number of strains analyzed	Codon	Nucleotide change	Amino-acid change	No. of strains with mutations
*rpoB*	35	513	CAA → GAA	gln → glu	1
		526	CAC → GAC	his → asp	1
		531	TCG → TTG	ser → leu	7
		533	CTG → CCG	leu → pro	1
*katG*	7		No mutations		0
*inhA*	7		C (−15) T		2
*gyrA*	5	94	GAC → GGC	asp → gly	1
		95	AGC → ACC	ser → thr	3

Sanger sequencing results of the *katG* gene and *inhA* promoter for the 7 MTB strains resistant to isoniazid. While no mutations were found in *katG*, 2 MTB strains (28.6%) had mutations in *inhA* (Table 2). In 5 MTB strains (71.4%) that were phenotypically isoniazid-resistant, there were no mutations found in either in *katG* or *inhA*.

Sanger sequencing results of the *rpoB* gene for the 5 MTB strains resistant to fluoroquinolones are shown in Table 2. One MTB strain (20%) was discarded due to poor sequencing quality. For the remaining 4 MTB strains, all of these (100%) had mutations in *gyrA*. The four MTB strains had mutations in codon 95, and one of them also had a mutation in codon 94 (Table 2).

### The population structure of MTB isolates from Ibadan (Nigeria) included in this study

The population structure for the 51 MTB isolates included in the genotypic analysis is shown in the neighbor-joining tree (NJT) in Figure 1. Lineage identification using similarity search in MIRU-VNTRplus showed that 96.1% (49/51) of the strains belong to lineage 4 (L4), distributed as follows: Uganda I (24/51, 47.1%), LAM (11/51, 21.6%), Cameroon (9/51, 17.6%), and Ghana (5/51, 9.8%). Meanwhile, 3.9% (2/51) of the strains correspond to lineage 5 (L5), specifically the West African-1 sub-lineage. The population structure was very heterogeneous, and no active transmission clusters were detected through 12-MIRU-VNTR typing (Figure 1).

**Figure 2:**
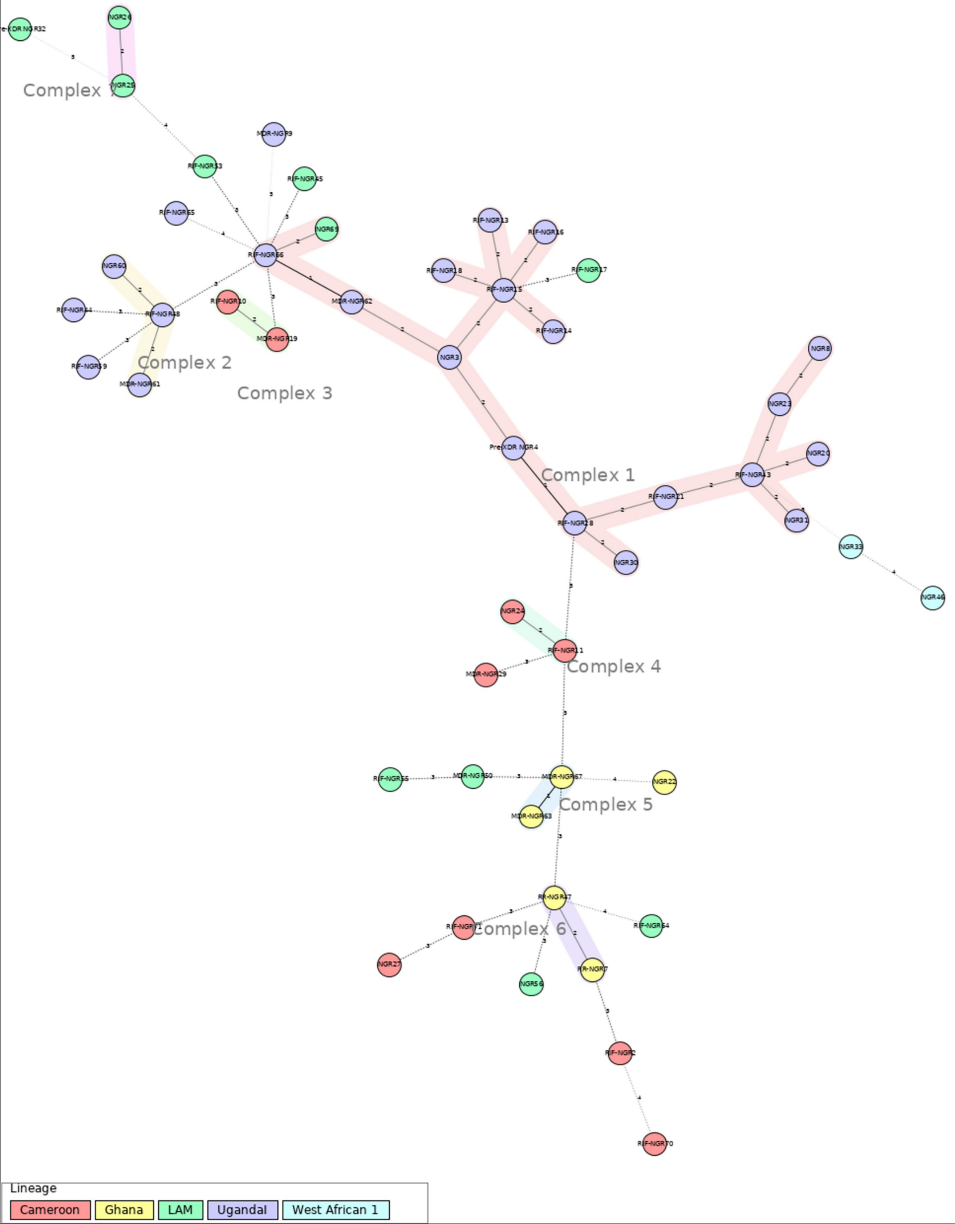
Minimum spanning tree (MST), based on 12-loci MIRU-VNTR analysis for the 51 MTB strains for Ibadan (Nigeria) included in this study. Genotypic lineages are distinguished by various colored dotted circles. The maximum locus difference between clonal complexes (CCs) is 2. The analysis was performed using the MIRU-VNTRplus web application.

The minimum spanning tree (MST) was done with a maximum difference of two loci for the detection of clonal complexes. This analysis shows 7 well-defined clonal complexes (Figure 2). Complex 1 consists of 18 MTB strains. Within this complex, there is a predominance of the Uganda I sublineage, and one strain has been identified as belonging to the LAM sublineage. Complexes 2 to 7 are made up of 2 to 3 strains. And 20 strains were singletons.

In Supplementary Table 2, the drug susceptibility profiles across the different MTB sublineages are detailed. The drug resistance MTB strains are concentrated in lineage 4, especially in strains from the sublineage Uganda I.

## Discussion

Nigeria has the highest tuberculosis infection rate in Africa, with 268 deaths reported daily. In 2020, the year this study’s samples were collected, the WHO recorded 138,591 TB cases in the country (a 15% increase from the previous year) (19). Despite this alarming situation, Nigeria has one of the world’s lowest TB detection rates, with only 24% of cases reported to the National Tuberculosis and Leprosy Control Program, further hindering efforts to combat the disease (20).

Our results for the mutation analysis of drug resistance genes are similar to previous report from Nigeria. A previous study from Ibadan reported a rifampicin resistance prevalence of 23.3%, closely aligning with the 28.6% observed in our study (21). Notably, missense mutations were identified, with the most prominent occurring at codon 531, as we also found (22). This mutation confers high-level rifampicin resistance by binding to the RNA polymerase subunit and inhibiting DNA-directed RNA synthesis in *M. tuberculosis* (22, 23). Moreover, mutations at codons 513, 526, and 533 found in our study have also been previously reported in Nigeria (24) and are associated with low-level rifampicin resistance (25).

Analysis of a *gyrA* gene fragment for fluoroquinolone resistance revealed a 60% incidence of the Ser95Thr mutation at codon 95 and a 20% occurrence of the Asp94Gly mutation at codon 94. Previous studies worldwide, including Nigeria, have already shown that mutations at codons 94 and 95 are the most common (26–28).

The C (−15 T) mutation in the *inhA* promoter, detected in 20% of strains, is a well-established marker for MDR-TB. Along with three other mutations (−16A > G, −8 T > A, and −8 T > C), it contributes to resistance by upregulating *inhA* expression (27). A similar study conducted in Southwestern Nigeria (28) has already reported that 89.7% of samples with phenotypic isoniazid resistance carried at least one known resistance-associated mutation. In contrast, our study did not identify any mutations within the amplified region of *katG*, suggesting that resistance may stem from mutations outside the analyzed section (29). Notably, up to 20% of isoniazid-resistance mutations occur outside *katG*, often in the *ahpC* gene (30, 31). Additionally, a significant proportion of clinical isolates exhibit resistance that cannot be fully explained by mutations in the analyzed genes, suggesting the involvement of alternative mechanisms such as efflux pump overexpression (32). *M. tuberculosis* contains multiple efflux pumps capable of extruding different classes of antibiotics. These multidrug resistance efflux pumps and transporters may be overexpressed due to mutations in regulatory regions, or their expression can be induced by antibiotic exposure through interactions with bacterial regulatory systems (33, 34). Furthermore, during *M. tuberculosis* infection, certain bacterial subpopulations can develop phenotypic tolerance to antimycobacterial drugs without acquiring genetic mutations (35). Another plausible cause is the presence of mutations located outside the routinely screened regions, such as outside the RRDR in the *rpoB* gene, which have been associated with low-level rifampicin resistance and may escape detection by standard molecular assays (e.g., I491 > F and V170 > F) (36). Moreover, heteroresistance, the coexistence of resistant and susceptible bacterial subpopulations within the same sample, has been increasingly recognized and may significantly contribute to phenotypic–genotypic discordance (37).

MIRU-VNTR analysis in this study identified L4 as the most prevalent lineage (96.1%), encompassing sub-lineages such as LAM, Ghana, Uganda I, and Cameroon. Previous studies conducted in various Nigerian states (8, 38), have also reported L4 as one of the most common lineages. However, the distribution of sublineages within L4 differs between different locations and study periods in Nigeria. For instance, a previous study from 2012 in Ibadan did not detect Uganda I, the most frequent sublineage in our study (39). However, another study done in Lagos reported a prevalence of 11.5% for Uganda I (30). On the other hand, the Cameroon sublineage has a lower prevalence in our study compared to previous reports from Nigeria (39, 40). Overall, these results highlight the significant variability of MTB sublineages over recent years and across different areas of Nigeria, likely fueled either by active transmission in certain settings and also by TB relapse in a highly exposed population across the country, as it has been reported in other settings (41–43). In fact, the lack of active transmission clusters within our study population at a low-resolution level, like 12 loci MIRU-VNTR supports the hypothesis of a very high diversity of MTB strains within Ibadan and the role of TB relapse in the regional epidemiology of TB in this location (41–43). Nevertheless, the diversity of sublineages and the lack of clustering may also be influenced by the limitations of the MIRU-VNTR technique itself, as it is not a perfect method for genotyping and is affected by homoplasy events in these molecular markers (12, 44). Therefore, MIRU-VNTR should be complemented with other methods, such as SNP analysis, spoligotyping, or WGS, to enhance analytical accuracy and improve lineage designation when possible.

The high burden of multidrug-resistant tuberculosis (MDR-TB) in developing countries such as Nigeria reflects systemic weaknesses in both diagnostic capacity and access to effective treatment compared to developed countries. In many low-income settings, limited availability of rapid resistance testing tools, such as GeneXpert MTB/RIF, delays appropriate therapy and contributes to the continued transmission of resistant strains (45). Common resistance-conferring mutations in *rpoB*, *katG*, and *inhA* genes, which are associated with resistance to rifampicin and isoniazid, are prevalent in Nigerian isolates, further complicating treatment and reflecting local selective pressures resulting from inconsistent therapy and informal antibiotic use.

The population structure in Nigeria, characterized by widespread and heterogeneous latent TB infection, contrasts with the localized, risk-group-driven patterns seen in high-income countries. This facilitates the sustained transmission and reactivation of diverse, often resistant *MTB* lineages (46). As a result, control strategies that focus primarily on interrupting transmission chains may be insufficient. Effective TB control in Nigeria requires an integrated approach that combines molecular surveillance, mutation-informed treatment protocols, and interventions targeting social determinants such as poverty and malnutrition (47). This highlights the need for comprehensive public health interventions that go beyond biomedical solutions and target the social conditions underpinning TB vulnerability.

This study emphasizes the implementation of cost-effective molecular approaches for the surveillance of drug-resistant *M. tuberculosis*, particularly resistance to first-line drugs, in resource-constrained settings. Overall, our results for mutation analysis of key antibiotic resistance genes support its use as an affordable method to address drug resistance analysis of MTB in the absence of WGS. However, a larger panel of target genes should be considered for a more sensitive detection of resistant strains. Locally, in Nigeria, where TB burden is high and public health budgets are limited, the adoption of in-house PCR protocols and targeted sequencing offers a practical solution to improve early detection of resistance and guide timely treatment decisions provides a feasible solution for improving early detection, enabling better tracking of transmission dynamics and drug resistance patterns, and informing timely treatment decisions (45, 48). Regionally, across Africa, where similar challenges in laboratory infrastructure persist, these scalable tools can support the identification of outbreaks, monitoring of treatment failures, and refinement of national control strategies (49). Globally, the deployment of affordable molecular genotyping contributes to enhanced surveillance and a better understanding of resistance evolution, supporting the WHO End TB Strategy by enabling data-driven responses in high-burden regions. Thus, the approaches proposed in this research offer a viable path forward for TB control at multiple levels of intervention (50).

One of the main limitations of this study is the relatively small sample size (*n* = 51), obtained from a single center, which may restrict the representativeness of the findings. Therefore, the results should be interpreted with caution and cannot be generalized to the broader population. Moreover, the analysis was limited to four resistance-associated genes, which may not fully capture the genetic basis of phenotypic resistance observed in some isolates. Future studies should include additional loci such as *embB*, *pncA*, *rpsL*, and *rrs*, in order to provide a more comprehensive understanding of drug resistance in the studied population. Also, whole genome sequencing should be considered in future studies for a deeper characterization of MTB isolates.

## Conclusion

Continuous epidemiological monitoring of *Mycobacterium tuberculosis* in Nigeria is essential, particularly given the country’s dense population, challenging socioeconomic conditions, and high levels of human mobility. Ongoing surveillance is vital for tracking the prevalence, distribution, and transmission dynamics of tuberculosis. It supports the identification of outbreaks, the design of more effective prevention and treatment strategies, the detection of drug resistance, and the evaluation of intervention effectiveness. Moreover, given the high burden of TB in Nigeria, there is an urgent need for further studies to understand better and address the challenges posed by the disease. In this sense, implementing affordable methods for monitoring drug resistance mutations monitoring, and MTB population dynamics would help to improve TB surveillance and control in a context where WGS is still an economical challenge.

## Data Availability

The original contributions presented in the study are included in the article/Supplementary material, further inquiries can be directed to the corresponding author.

## Footnotes

^1^ http://www.miru-vntrplus.org

^2^ https://www.ncbi.nlm.nih.gov/genome/
