# Supplementary material for: Population structure and antibiotic resistance profiles of Mycobacterium tuberculosis isolates from Ibadan, Nigeria (2019–2020): a pilot study to improve affordable molecular diagnostic tools
Source: Front Public Health. 2025 Sep 29;13:1657825. doi: 10.3389/fpubh.2025.1657825 (PMC12515896; doi:10.3389/fpubh.2025.1657825)
Supplement: Supplementary file 1 [file Data_Sheet_1.pdf]

Supplementary Table 1. PCR primers for *rpoB*, *katG*, *gyrA*, and the *inhA* promoter.

Target	Sequence 5'- 3'	Amplified region	Amplicon size (bp)	Annealing temperature	References
rpoB	F: CGTGGAGGCGATCACACCGCAGACGT<br>R: AGTGCGACGGGTGCACGTGCGGGACCT	493-563	210	65 °C	(11)
katG	F: CGTGGAGGCGATCACACCGCAGACGT<br>R: AGTGCGACGGGTGCACGTGCGGGACCT	300-505	620	55 °C	(11)
gyrA	F: ATCGAGCAGGAGATGCA<br>R: CGTCGTAGTTAGGGATGAA	2364-2777	414	55 °C	(9)
inhA promoter	F: CCTCGCTGCCCAGAAAGGGA<br>R: ATCCCCCGGTTTCCTCCGGT	-168 to -149<br>47-66	249	55 °C	(9)

Supplementary Table 2. Drug resistance profiles distribution across the MTB sublineages found in this study (RIF=rifampicin; INH=izoniazid; FQ=fluoroquinolones; MDR=multidrug resistant; pre-XDR=pre-extensively drug-resistant).

Sublineage	RIF	INH	FQ	MDR (RIF+INH)	MDR (RIF+FQ)	Pre-XDR (RIF+INH+FQ)	Sensible
LAM	5	-	-	1	-	1	4
Uganda I	13	-	-	2	1	1	7
Cameroon	5	-	-	-	2	-	2
Ghana	2	-	-	-	2	-	1
West African 1	-	-	-	-	-	-	2
**Total**	25	0	0	3	5	2	16
